# Biologic treatments for psoriasis have different anatomical specificities in residual PASI

**DOI:** 10.1111/ddg.15924

**Published:** 2025-10-11

**Authors:** Martina Burlando, Irene Schiavetti, Aurora Parodi, Emanuele Cozzani, Luca Mastorino, Eleonora Bongiovanni, Ilaria Salvi, Martina Del Vecchio, Paolo Dapavo

**Affiliations:** ^1^ Clinica Dermatologica, DissaL, Università di Genova Ospedale Policlinico San Martino – IRCCS Genova Italy; ^2^ Section of Biostatistics Department of Health Sciences University of Genova Genova Italy; ^3^ Section of Dermatology Department of Medical Sciences University of Turin Turin Italy

**Keywords:** Biologics, Psoriasis, residual PASI

## Abstract

**Background and objectives:**

Improved understanding of the immunopathogenesis of psoriasis has led to the development of effective targeted therapies, but patients who respond to treatment without total skin clearance have residual disease. The aim of this study was to demonstrate the existence of specific residual disease (or residual PASI) anatomical localizations related to different biologic agents.

**Methods and patients:**

This retrospective, observational, multi‐center study analyzed clinical data of patients affected by psoriasis who had received biologic treatments for at least 6 months. The data analysis focused on patients with residual PASI.

**Results:**

A total of 228 of 1,000 patients showed residual disease despite achieving PASI 90 at weeks 24–28 of biologic treatment. The anatomical sites most frequently involved in residual disease were the lower limbs (44.3%). We observed differences among the biologic agents in terms of frequency and localization of residual disease. The localization of residual skin lesions to lower limbs was associated with treatment switching/interruption. The drugs with the highest and lowest proportion of patients with residual disease in the lower limbs were, respectively, secukinumab and risankizumab.

**Conclusions:**

Treatment with anti‐IL‐17 and anti‐IL‐23 drugs is characterized by the persistence of residual skin lesions with differences in terms of frequency and anatomical localizations.

## INTRODUCTION

Psoriasis is a chronic inflammatory skin condition that affects 2%–3% of the global population.^1^, ^2^ It is characterized by the uncontrolled activation of both adaptive and innate immunity, which leads to an overproduction of pro‐inflammatory cytokines such as tumor necrosis factor (TNF)‐α and interleukin (IL)‐17.^3^ While advancements in treatment have allowed many patients to achieve near‐complete skin clearance,^4^ those who do not attain total skin clearance (TSC) often experience negative impacts on their health‐related quality of life (HRQoL) and an increased risk of comorbidities, such as psoriatic arthritis.^5^ Understanding the specific locations of residual disease associated with different biologic agents can help guide treatment decisions effectively.

## MATERIALS AND METHODS

This observational study analyzed data from patients with psoriasis who were referred to the Dermatology Departments of Policlinico San Martino Hospital and the University of Turin, Italy, during 2022. A total of 1,000 patients received either anti‐IL‐17 treatments (secukinumab, ixekizumab, brodalumab, bimekizumab) or anti‐IL‐23 treatments (guselkumab, risankizumab, tildrakizumab) for at least 6 months. The focus was on patients with residual Psoriasis Area Severity Index (PASI) after treatment, examining skin lesion localization and various outcome measures, including the timing of achieving PASI 90.

## RESULTS

Out of the 1,000 patients, 228 exhibited residual lesions after treatment, while 772 achieved PASI 100 and were therefore excluded from the study. Among those with residual lesions, 61.8% had comorbidities, and the mean age at diagnosis was 38.6 years. Demographic data, habits and comorbidities are summarized in Table 1.

**TABLE 1:** Demographic data, habits, and comorbidities (n = 228).

**Ethnicity**	Caucasian	218 (95.6%)
	Black	3 (1.3%)
	Other	7 (3.1%)
**Sex**	Female	82 (36.0%)
	Male	146 (64.0%)
**Age, years**		53.3 ± 15.34 (19.0 – 89.0)
**BMI**		26.4 ± 4.94
**Alcohol**	Never used alcohol	62 (27.2%)
	Occasional consumption	99 (43.4%)
	Regular consumption	26 (11.4%)
	Not specified	41 (18.0%)
**Smoke**	Never smoked	71 (31.1%)
	Former smoker	58 (25.4%)
	Smoker	99 (43.4%)
**Comorbidities**		
	At least one comorbidity	141 (61.8%)
	Autoimmune disease	Untreated 1 (0.4%) Treated 9 (3.9%)
	Cerebrovascular disease	Untreated 0 (0.0%) Treated 5 (2.2%)
	Cardiovascular disease	Untreated 1 (0.4%) Treated 45 (19.7%)
	Chronic kidney disease	Untreated 0 (0.0%) Treated 2 (0.9%)
	Gastrointestinal disease	Untreated 5 (2.2%) Treated 11 (4.8%)
	HIV	Untreated 0 (0.0%) Treated 0 (0.0%)
	HBV	Untreated 1 (0.4%) Treated 5 (2.2%)
	Hematological disease	Untreated 3 (1.3%) Treated 5 (2.2%)
	Hypertension	Untreated 2 (0.9%) Treated 62 (27.2%)
	Malignant tumor	Untreated 0 (0.0%) Treated 12 (5.3%)
	Metabolic disease	Untreated 6 (2.6%) Treated 53 (23.2%)
	Musculoskeletal disease	Untreated 2 (0.9%) Treated 11 (4.8%)
	Neurologic disease	Untreated 0 (0.0%) Treated 2 (0.9%)
	Psychiatric disease	Untreated 0 (0.0%) Treated 8 (3.5%)
	Other	Untreated 5 (2.2%) Treated 31 (13.6%)

**FIGURE 1:**
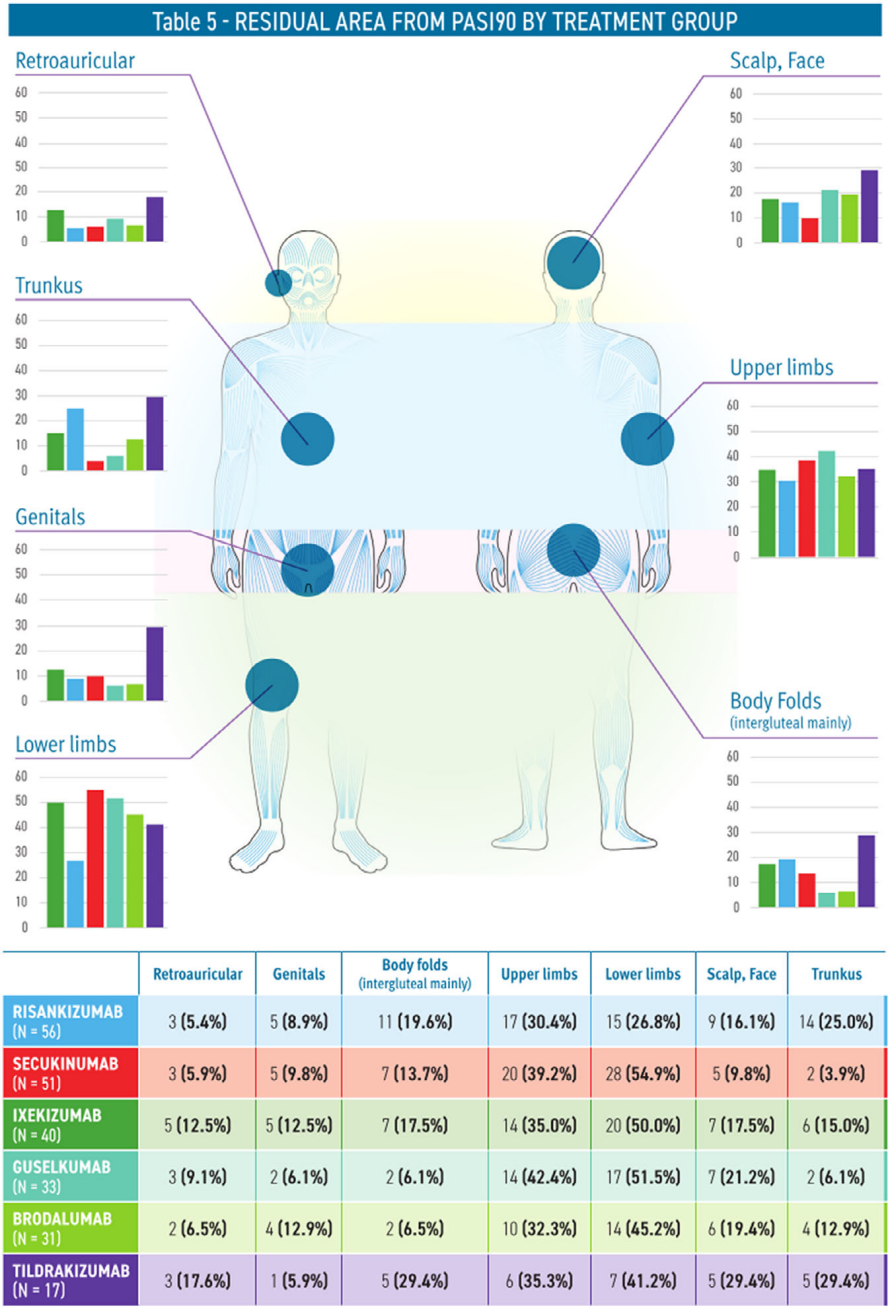
Residual areas from PASI 90 by treatment group.

A PASI 90 response was achieved after an average of 19.5 weeks. The most affected areas included the lower limbs (44.3%), upper limbs (35.5%), and scalp/face (17.1%). Notably, secukinumab exhibited the highest incidence of residual disease in the lower limbs (54.9%), while risankizumab and tildrakizumab were associated with a higher prevalence of lesions on the trunk (Figure 1).

Seventeen percent of patients changed their treatment, primarily at the recommendation of their physician (84.6%). Lower limb lesions were associated with a higher rate of treatment modifications (22.8%). Brodalumab and risankizumab were the most frequently chosen options for switching treatments. Factors influencing these therapy switches included previous treatments with secukinumab and ixekizumab. Interestingly, patients rated their acceptance of residual lesions at 83.1 ± 17.88, with 85.0% stating that residual disease did not prompt a change in medication. The clinical characteristics and management of patients who achieved PASI 90 are summarized in Table 2.

**TABLE 2:** Clinical characteristics and management of patients who achieved PASI 90.

PASI at the beginning of the last treatment		14.8 ± 5.90 (5.0–37.0)
PGA at the beginning of last treatment	Clear	0 (0.0%)
	Almost clear	1 (0.4%)
	Mild	4 (1.8%)
	Moderate	85 (37.3%)
	Moderate to severe	95 (41.7%)
	Severe	43 (18.9%)
Concomitant topical treatment	No topical	150 (65.8%)
	Clobetasol foam	7 (3.1%)
	Clobetasol + Calcipotriol foam	56 (24.6%)
	Clobetasol + Calcipotriol gel	15 (6.6%)
Week of treatment at which PASI 90 is achieved		19.5 ± 9.53 (4.0 ‐ 28.0)
PGA when PASI 90 is achieved	Clear	161 (70.6%)
	Almost clear	64 (28.1%)
	Mild	2 (0.9%)
	Moderate	0 (0.0%)
	Moderate to severe	1 (0.4%)
	Severe	0 (0.0%)
Residual area	Retroauricular	19 (8.3%)
	Genitals	22 (9.6%)
	Folds (intergluteal mainly)	34 (14.9%)
	Upper limbs	81 (35.5%)
	Lower limbs	101 (44.3%)
	Scalp, face	39 (17.1%)
	Trunk	33 (14.5%)
Therapy switched or interrupted		39 (17.1%)
For whose primary choice?	Physician	33 (84.6%)
	Patient	6 (15.4%)
New therapy	Brodalumab	10 (25.6%)
	Risankizumab	9 (23.1%)
	Ixekizumab	8 (20.5%)
	No therapy	5 (12.8%)
	Bimekizumab	2 (5.1%)
	Certolizumab pegol	1 (2.6%)
	Secukinumab	1 (2.6%)
	Guselkumab	1 (2.6%)
	Tildrakizumab	1 (2.6%)
	Ustekinumab	1 (2.6%)
How much does the patient accept his or her residual PASI condition?		83.1 ± 17.88 (5.0–100.0)
Does the residual PASI at that site motivate the patient to change medication?	No	192 (85.0%)
	Yes	34 (15.0%)

## DISCUSSION

Our study is the first to investigate the relationship between individual biological drugs in the anti‐IL‐17 and anti‐IL‐23 classes and the locations of residual disease (residual PASI) after achieving PASI 90. We found that secukinumab had the highest prevalence (over 54%) of residual disease in the lower limbs, which was significantly associated with the switch or interruption of treatment. Ixekizumab and brodalumab followed with 50.0% and 45.2%, respectively, while risankizumab and tildrakizumab showed the lowest prevalence (26.8% and 41.2%).

The lower rates of residual disease with risankizumab and tildrakizumab may be related to the role of IL‐23 in T helper cell differentiation.^6^ Interestingly, residual PASI localized to the lower limbs was the only anatomical factor associated with a higher odds of treatment modification, although previous data suggest that residual lesions in more visible areas negatively affect patient‐reported outcomes.^7^ This may indicate that patients with lower limb lesions maintain a significant disease burden even with PASI 90.

Mashiko et al. found that residual plaques, although responding well to treatment, still show molecular differences from untreated plaques, indicating the presence of key psoriasis pathways and resistance to resolution.^8^

In our study, 85% of patients indicated that residual PASI would not prompt them to change medications, and most accepted the persistence of their disease. This contrasts with other reports indicating that even minimal skin lesions can adversely affect quality of life, as reflected by *Dermatology Life Quality Index* (DLQI) scores greater than 1, with 20% of patients who achieved complete skin clearance still reporting a negative impact of psoriasis.^7^

One‐third of patients in our study were bionaïve, which can lead to improved treatment effectiveness. Randomized controlled trials on IL‐17/IL‐23 inhibitors have shown better patient‐reported outcomes among bionaïve individuals.^7^

Interestingly, nearly 85% of treatment changes were made by physicians, possibly highlighting a misalignment between clinicians' and patients' perceptions of disease impact since most patients accepted residual skin lesions.

Patients achieved a ≥ 90% improvement in PASI score after a median of 19.5 weeks, with variability among biologic agents. The speed of action is crucial for patients with acute flare‐ups, while those with a stable history may accept slower treatments for better safety.

Our study focused on patients with residual PASI, which may have introduced selection bias and limited representativeness, as it was conducted in only two university centers in Italy. While retrospective data collection was a limitation, the findings can be valuable for integrating outcomes related to residual skin lesions into clinical guidelines practice.

## CONFLICT OF INTEREST STATEMENT

M.B. has acted as a speaker for AbbVie, Almirall, Amgen, Eli Lilly, Janssen, Novartis, and UCB. A.P. has acted as a speaker for AbbVie, Almirall, Eli Lilly, Janssen, Novartis, and UCB. E.C. has acted as a speaker for AbbVie, Almirall, Eli Lilly, and Novartis. L.M. has acted as a speaker for AbbVie, Leo Pharma, Accord, and Almirall. P.D. has acted as a speaker for AbbVie, Almirall, Amgen, Eli Lilly, Janssen, Novartis, and UCB.
